# Identification of potential long non-coding RNA biomarkers associated with the progression of colon cancer

**DOI:** 10.18632/oncotarget.17924

**Published:** 2017-05-17

**Authors:** Jingwen Li, Weinan Xue, Junli Lv, Peng Han, Yanlong Liu, Binbin Cui

**Affiliations:** ^1^ Department of Colorectal Surgery, Harbin Medical University Cancer Hospital, Harbin 150081, China; ^2^ Department of Science, Harbin Medical University Cancer Hospital, Harbin 150081, China

**Keywords:** biomarkers, colon cancer, disease progression, long non-coding RNA

## Abstract

Increasing evidence has suggested that dysregulated lncRNA expression played important roles in the development and progression of human cancers. Although prognostic roles of lncRNAs have been recognized for colon cancer (CC) patients, the search for novel lncRNA biomarkers potentially involved in CC progression is an urgent and still largely unmet medical need. In this study, we evaluated the lncRNA expression changes during the progression of CC by analyzing two cohorts of previously published expression profiles of CC patients and identified hundreds of differentially expressed lncRNAs. Then we identified eight lncRNAs that closely associated with the progression of CC patients from a large number of significantly altered lncRNAs using random forest supervised classification algorithm. Finally, an SVM-based lncRNA risk classifier was developed to discriminate high-risk CC patients from persons with early-stage and validated in both the training dataset and testing dataset by survival analysis and five-fold cross-validation strategy. Our pathway enrichment analysis based on protein-coding genes that are co-expressed with lncRNAs, suggested that variation in expression of eight lncRNAs biomarkers might affect critical pathways involved in CC progression. With further validation, these eight lncRNAs might have significant implications for the clinical management of CC patients with early stage and improve our understanding of cancer progression.

## INTRODUCTION

Colon cancer (CC) is one of the most common types of human cancers and is the major cause of cancer-related death worldwide [1]. Surgery followed by adjuvant therapy (such as chemotherapy and radiation therapy) is the most common option for CC patients. However, the fact that early-stage CC patients with an initial treatment with surgery are known to still have a recurrence rate of 20%–30% and are likely to progress into malignancy [2]. Systemic treatment of early-stage patients with high-risk for disease progression attempts to prevent disease progression and improve patients’ outcome. Therefore, it is an urgent need to identify high-risk patients who are likely to progress into malignancy at an early stage of cancer development.

With recent advances in high-throughput technologies (such as RNA deep sequencing), transcriptomes of many organisms have been surveyed which identified thousands of long transcripts (> 200 bp) that have no significant protein-coding capacity and thus termed long noncoding RNAs (lncRNAs) [3]. Similar to mRNAs and microRNAs, increasing evidence has suggested that lncRNA are the key regulators of transcriptional and translational output and therefore make effects in many biological and pathological processes (such as dosage compensation, genomic imprinting, cell differentiation and organogenesis) at the epigenetic level, transcriptional and post-transcriptional level [4, 5]. There is generally believed that lncRNAs are emerging as a novel hallmark of cancer beyond ten underlying principles shared by all cancers [6]. A large number of lncRNAs are found to be differentially expressed in various types of human cancer. Recently many studies have demonstrated their intriguing possibilities of application for diagnostics, prognostics and therapeutics in different cancers, including breast cancer, lung cancer, ovarian cancer, multiple myeloma and so on [7–23]. Although prognostic roles of lncRNAs have been recognized for CC patients, the search for novel lncRNA biomarkers potentially involved in CC progression is an urgent and still largely unmet medical need.

The aim of the present study was to evaluate lncRNA expression changes during the progression of CC by analyzing two cohorts of previously published expression profiles of CC patients, and tried to identify specific lncRNAs biomarkers closely associated with the progression of CC patients from a large number of significantly altered lncRNAs.

## RESULTS

### Identification of altered lncRNAs in the progression of LC

The GSE37892 patient dataset was chosen as a training dataset for the detection of lncRNA biomarkers. We first compared lncRNA expression profiles among patients with early-stage and those with advanced-stage in the training dataset, and identified 422 differentially expressed lncRNAs (FDR-adjusted *p* ≤ 0.01) using the SAM analysis. Of them, 269 lncRNAs are up-regulated and 153 lncRNAs are down-regulated in patients with advanced-stage compared with early-stage patients.

**Figure 1:**
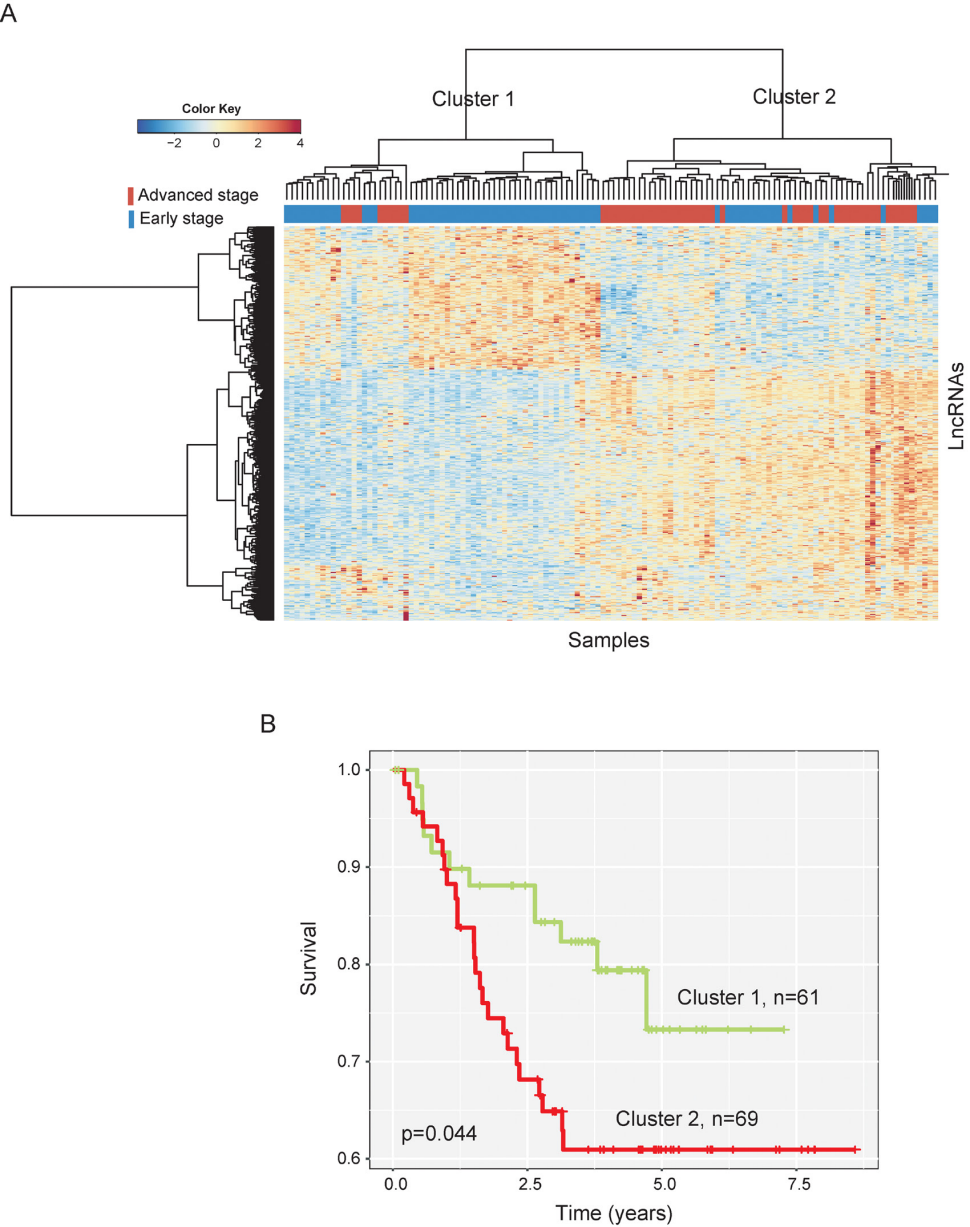
The heatmap and survival analysis of hierarchical clustering of 130 CC patients of the training dataset based on differentially expressed lncRNAs (**A**) Heatmap of differentially expressed lncRNAs between early-stage and advanced-stage breast cancers. (**B**) Kaplan-Meier survival curves of metastasis-free survival between two clusters.

Hierarchical clustering of these 422 lncRNAs based on centered Pearson correlation clearly separated advanced-stage patients from early-stage patients (Figure 1A). Only 33 patients (10 advanced-stage patients and 22 early-stage patients) were misclassified by the clustering analysis. Cluster 1 consisted of 61 patients, including 51 early-stage patients and 10 advanced-stage patients, whereas cluster 2 consisted of 69 patients, including 22 early-stage patients and 47 advanced-stage patients, which achieved a high prediction accuracy of 74.6%. The statistical result suggested that two patient clusters grouped by these 422 lncRNAs were significantly correlated with disease progression status (*p* < 0.001, Chi-square test). The Kaplan-Meier curves and log-rank test showed that metastasis-free survival (MFS) was significantly different between these two patient clusters (*p* = 0.044, log-rank test) (Figure 1B).

### Identification of optimal lncRNA biomarkers significantly associated with the progression of CC from the training dataset

**Figure 2:**
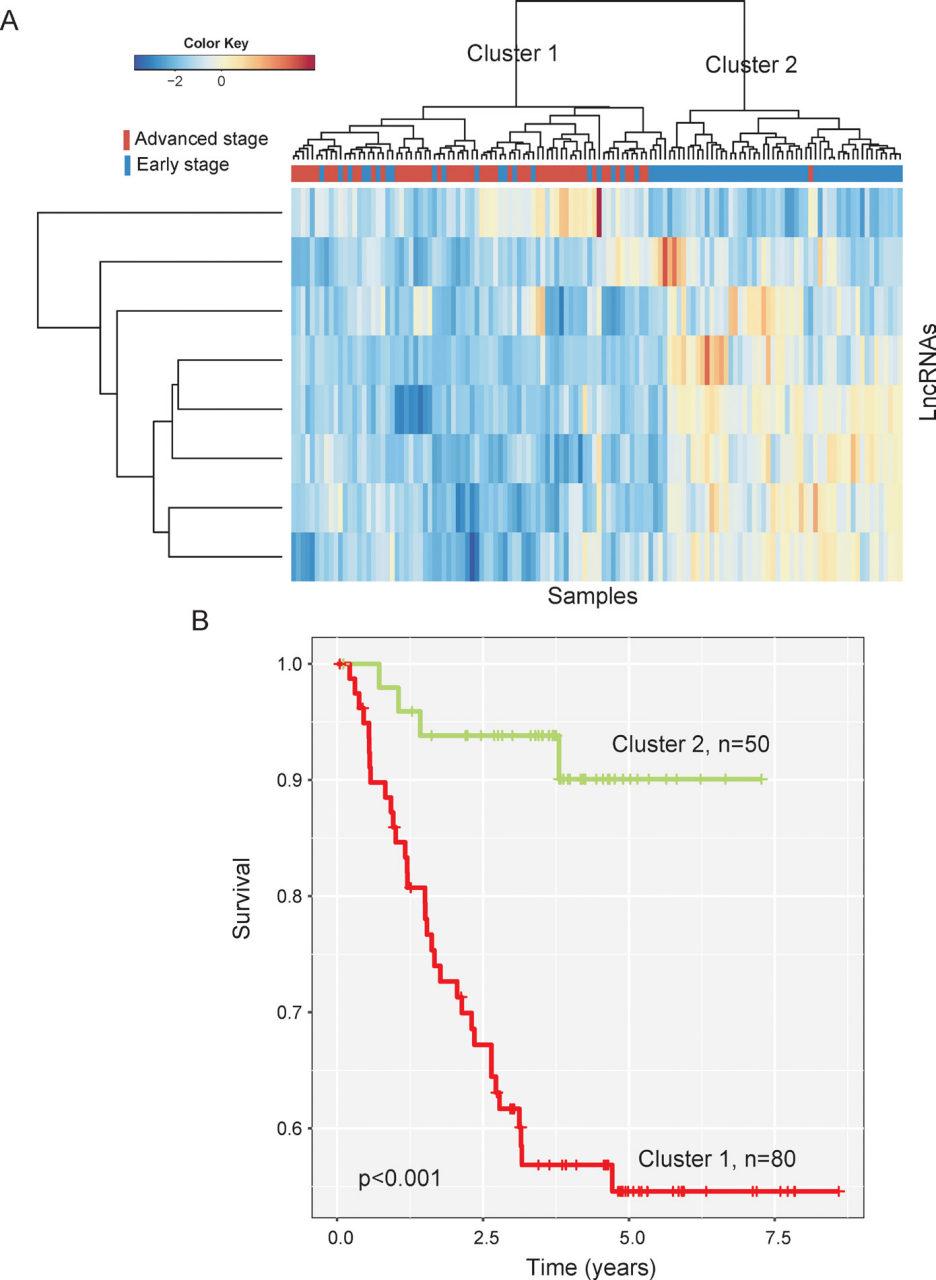
The heatmap and survival analysis of hierarchical clustering of 130 CC patients of the training dataset based on selected eight lncRNA biomarkers (**A**) Heatmap of selected eight lncRNA biomarkers. (**B**) Kaplan-Meier survival curves of metastasis-free survival between two clusters.

In order to identify optimal lncRNA biomarkers for clinical use, a random forest supervised classification algorithm was used to narrow down the number of lncRNAs using these differentially expressed lncRNAs as features and their expression levels as feature values. Finally, eight lncRNAs mostly related to the prognostic classification were selected as optimal biomarkers among 422 differentially expressed lncRNAs according to the permutation important score (Table 1). Hierarchical clustering analysis with selected eight lncRNA biomarkers clearly separated the 130 patients of training dataset into two clusters (Figure 2A). Cluster 1 consisted of 80 patients, including 56 advanced-stage patients and 24 early-stage patients, whereas cluster 2 consisted of 50 patients, including 49 early-stage patients and only one advanced-stage patient, which achieved a high prediction accuracy of 80.8%. The Kaplan-Meier curves and log-rank test showed that MFS was significantly different between these two patient clusters grouped by these eight lncRNA biomarkers (*p* < 0.001, log-rank test) (Figure 2B). These results revealed the better predictive performance of eight lncRNA biomarkers for identifying patients at high risk of CC progression

**Table 1:** Eight lncRNA biomarkers associated with the progression of colon cancer

Ensembl id	Gene name	Chromosomal location	FC	FDR
ENSG00000232560	LINC01549	Chr21: 17,438,890-17,449,185 (+)	0.827	0
ENSG00000232656	IDI2-AS1	Chr10: 1,022,666-1,044,201 (+)	0.75	0
ENSG00000214188	ST7-OT4	Chr7: 116,953,899-117,098,806 (+)	0.929	0.002
LOC100505942	LOC100505942	Chr16: 67,517,706-67,528,745(−)	1.157	0
ENSG00000244791	RP11-65D17.1	Chr8: 126,325,495-126,329,535(+)	0.862	0
ENSG00000228391	AC011995.3	Chr2: 2,870,558-2,871,231(−)	0.83	0
ENSG00000264859	DSG2-AS1	Chr 18: 31,542,146-31,556,911(−)	0.791	0
ENSG00000240567	RP11-3P17.4	Chr3: 161,426,427-161,448,242 (+)	0.469	0

### SVM-based lncRNA risk classifiers distinguished advanced-stage and early-stage patients

**Figure 3:**
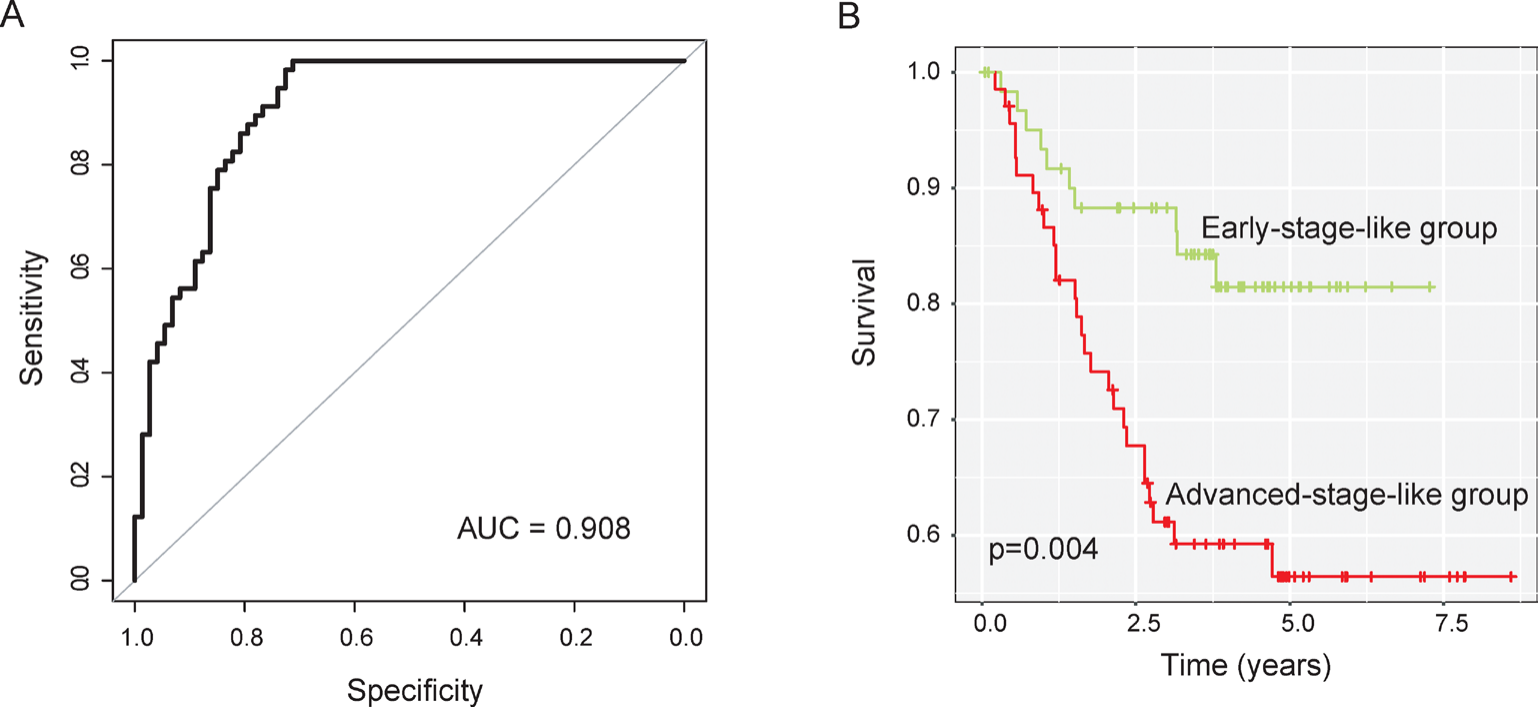
Performance evaluation of SVM-based lncRNA risk classifiers in the training dataset (**A**) Receiver operating characteristic (ROC) curves for SVM-based lncRNA risk classifiers in distinguishing advanced-stage and early-stage patients (**B**) Kaplan-Meier survival curves of metastasis-free survival between two early-stage-like group and advanced-stage-like group.

Subsequently, these eight lncRNA biomarkers were integrated into a risk classifiers using SVM to identify patients at high risk for progression. SVM analysis with a 5-fold cross-validation procedure was performed to evaluate the predictive performance of the SVM-based lncRNA risk classifiers. Results of performance evaluation showed that the SVM-based lncRNA risk classifiers were able to correctly classify 107 out of 130 patients, achieving an overall predictive accuracy of 85.4% with a sensitivity of 89.5% and a specificity of 76.7%. The AUC of ROC analysis is 0.908 (Figure 3A). The Kaplan-Meier analysis demonstrated a significant difference in MFS between predicted early-stage-like group and advanced-stage-like group (Figure 3B). Patients in predicted early-stage-like group have significantly higher MFS than those in the predicted advanced-stage-like group (*p* = 0.004, log-rank test). The three-year and five-year MFS rates of the predicted advanced-stage-like group were 59.2% and 56.4%, respectively, whereas the corresponding rates in the predicted early-stage-like group were 86.3% and 81.5%, respectively.

### Further validation of optimal lncRNA biomarkers in another independent testing dataset

**Figure 4:**
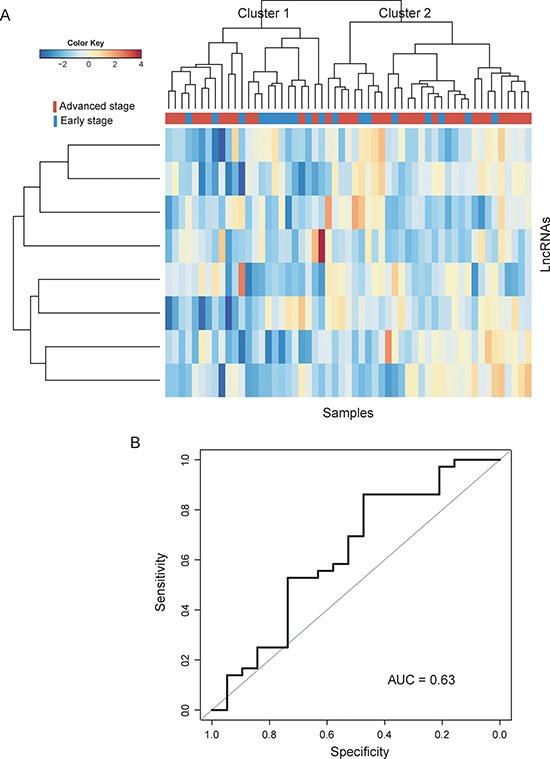
Performance validation of SVM-based lncRNA risk classifiers in the testing dataset (**A**) Heatmap of selected eight lncRNA biomarkers. (**B**) Receiver operating characteristic (ROC) curves for SVM-based lncRNA risk classifiers in distinguishing advanced-stage and early-stage patients.

To further confirm the predictive performance of optimal lncRNA biomarkers, eight lncRNA biomarkers were tested using another completely independent dataset of 55 patients from Smith's study. We first clustered CC patients in the testing dataset according to the expression value of eight lncRNA biomarkers. Results with unsupervised hierarchical clustering analysis were similar to that observed in the training dataset. Two distinctive patient clusters were obtained by hierarchical clustering analysis (Figure 4A). Cluster 1 consisted of 24 patients, including 13 advanced-stage patients and 11 early-stage patients, whereas cluster 2 consisted of 31 patients, including 8 early-stage patients and 23 advanced-stage patients, which achieved an overall prediction accuracy of 61.8%. Then the SVM-based lncRNA risk classifiers composing of eight lncRNA biomarkers in combination with 5-fold cross validation was validated in the testing dataset for determining patients at high-risk of disease progression. As expected, ROC analysis suggested that the AUC is 0.63 with a predictive accuracy of 63.6%, sensitivity of 65.4% and specificity of 33.3% (Figure 4B).

### Functional analysis of eight lncRNA biomarkers

**Figure 5:**
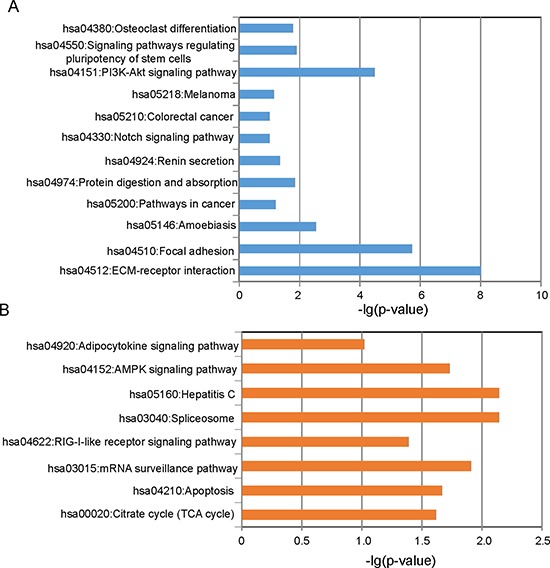
Functional enrichment analysis (**A**) Enriched KEGG pathways of protein coding genes positively correlated with lncRNAs. (**B**) Enriched KEGG pathways of protein coding genes negatively correlated with lncRNAs.

To better understand the functional roles of eight lncRNA biomarkers, we examined the expression correlation between their expression values and those of the mRNAs in the training dataset and identified protein-coding genes correlated with the eight lncRNA biomarkers. Finally, 1117 protein-coding genes were found to be positively correlated with at least one of the eight lncRNAs, and 1065 protein-coding genes were negatively correlated with at least one of the eight lncRNAs (top 1% as our criterion). Results of enrichment analysis suggested that 1117 protein coding genes positively correlated with lncRNAs mainly involved the following pathways: citrate cycle (TCA cycle), apoptosis, mRNA surveillance pathway, RIG-I-like receptor signaling pathway, spliceosome, Hepatitis C, AMPK signaling pathway and adipocytokine signaling pathway, and 1065 protein-coding genes negatively correlated with lncRNAs clustered most significantly in ECM-receptor interaction, focal adhesion, amoebiasis, pathways in cancer, protein digestion and absorption, renin secretion, notch signaling pathway, colorectal cancer, melanoma, PI3K-Akt signaling pathway, signaling pathways regulating pluripotency of stem cells and osteoclast differentiation (Figure 5).

## DISCUSSION

Despite recent significant advances in treatment and management of colon cancer patients, cancer recurrence following initial treatment with surgery remains a considerable problem. Almost half of all colorectal cancer patients will develop recurrent disease [24]. For those patients with stage III, the recurrence rate can exceed 50% and adjuvant chemotherapy has been shown to significantly reduce the risk of recurrence. Despite patients with stage I and II have more favorable outcomes with survival rates of 75% to 95% at 5 years, some patients with stage II faced an increased risk of recurrence approaching that of stage III colon cancer patients and chemotherapy is advised for those patients with stage II disease and reduce the risk of disease recurrence [25, 26]. However, adjuvant therapy, such as chemotherapy, has a wide range of side effects that substantially affect the patients’ life quality. Therefore, it is an urgent need to identify patients with early-stage colon cancer at high risk who might benefit from more aggressive therapy. Current clinic-pathologic information, such as TNM staging system, is inadequate for recurrence prediction of colon cancer patients with early-stage [25]. With the development in high-throughput gene expression and sequencing technologies, molecular profiles have been shown to be a powerful tool in diagnosis and prognosis of colon cancer, such as mRNAs and miRNAs [27, 28]. A recent study of ncRNAs has identified lncRNAs as a novel ncRNA class [3]. It is now evident that dysregulation of lncRNAs has been observed in the development and progression of many cancers, including colon cancer [10, 29–31]. Therefore, we attempt to identify potential lncRNA biomarkers of CC progression which could help determine patient with early-stage at the high-risk of disease recurrence and would allow selecting those patients for more aggressive treatment.

In this study, we performed a comprehensive analysis of lncRNA expression profiles of early-stage and advanced-stage CC patients and found altered lncRNA expression pattern during the progression of CC. Comparative analysis of lncRNA expression alternations between early-stage and advanced-stage CC patients improves our understanding of CC progression. Previous studies often focused on mRNAs and miRNAs, and identified altered mRNA/miRNA expression patterns during the progression of CC [27, 28, 32–35]. However, it is shown that lncRNAs are typically expressed in more cell-type-, and tissue-specific manner than mRNAs or miRNAs, thus having great advantages and priorities as diagnostic and prognostic markers [36]. Although lncRNA expression profiles have been investigated between CC patients and normal tissues and between cancer subtypes [37, 38], this is the first report that indicates the existence of stage-specific lncRNA expression pattern between early-stage and advanced-stage CC patients. Then we used bioinformatics methods to identify eight lncRNAs from a large number of significantly altered lncRNAs as potential biomarkers of CC progression. By performing hierarchical clustering, survival analysis and 5-fold cross-validation strategy, the predictive value of these eight lncRNAs was validated in the training dataset and testing dataset.

Although more and more lncRNAs have been discovered during the past years, a small fraction of lncRNAs has been functionally characterized. Functional study of individual lncRNAs still remains challenging [39]. Since current knowledge suggests that lncRNAs function by regulating or interacting with its partner molecules, identifying the function of protein-coding genes that are co-expressed with lncRNAs has been shown to be an effective way to better characterize the potential functions of novel lncRNAs. Our pathway enrichment analysis based on protein-coding genes that are co-expressed with lncRNAs, suggested that variation in expression of eight lncRNAs biomarkers might affect critical pathways involved in CC progression. For example, apoptosis-related genes were associated with high disease recurrence rates and have been used to identify stage II and III colon cancer patients with high risk of recurrence [40]. Perturbations of the AKT signaling pathway have been implicated in human cancers, including colon cancer. Overexpression of AKT was observed as an early event during colon tumorigenesis [41]. Focal adhesion kinase (FAK) expression has been correlated with worse prognosis in several tumors and may be involved in cancer radio- and chemosensitivity [42]. A component of focal adhesion, TRIM15, has been found to function as a tumor suppressor in colon cancer [43]. It is well known that Notch signaling is an important molecular pathway involved in the determination of cell fate. Genes of Notch signaling pathway such as Notch1, Notch2, HES1, DLL1, and JAG1 have been reported to be associated with the pathological tumor characteristics and degree of differentiation in colorectal cancer [44, 45].

In conclusion, our study has shown that the lncRNA expression pattern is altered in advanced-stage CC patients compared with early-stage CC patients. We identified eight lncRNAs as potential biomarkers capable of identifying patients with early-stage at high risk for progression to advanced-stage using differentially expressed analysis and random forest supervised classification algorithm methods. Finally, an SVM-based lncRNA risk classifier was developed to discriminate high-risk CC patients from persons with early-stage and validated in both the training dataset and testing dataset. With further validation, these eight lncRNAs might have important implications for the clinical management of CC patients with early-stage.

## MATERIALS AND METHODS

### Patient datasets

Two patient datasets of colon cancer were collected from the Gene Expression Omnibus (GEO) database ( https://www.ncbi.nlm.nih.gov/geo/). A total of 185 patients with CC was used in this study, including 130 CC patients (including 73 stage-II patients and 57 stage-III patients) in GSE37892 ( https://www.ncbi.nlm.nih.gov/geo/query/acc.cgi?acc=GSE37892) from Laibe's study and 55 CC patients (including 4 stage-I, 15 stage-II, 19 stage-III and 17 stage-IVpatients) in GSE17537 ( https://www.ncbi.nlm.nih.gov/geo/query/acc.cgi?acc=GSE17537) from Smith's study.

### Acquisition and analysis of lncRNA expression profiles

Raw gene expression data profiled from Affymetrix Human Genome U133 Plus 2.0 Array (HG-U133_Plus_2.0) in the three patient cohorts were processed and normalized using the Robust Multichip Average (RMA) algorithm for background adjustment and log-transformed (base 2). LncRNA expression profiles of CC patients in this study were obtained by repurposing the probes in the HG-U133_Plus_2.0 array according to Zhang's study [46]. Briefly, probes (probe sets) representing lncRNAs were obtained by remapping their RefSeq IDs and Ensembl IDs to the annotation of lncRNAs from GENCODE.

Significance analysis of microarrays (SAM) was applied to identify differentially expressed lncRNAs between patients with early-stage (stage I/II) and those with advanced-stage (stage III/IV). Those lncRNAs with FDR ≤ 0.01 (Benjamini and Hochberg's multiple-test adjustment) from SAM analysis were identified as differentially expressed lncRNAs. Unsupervised hierarchical clustering was used to investigate the effectiveness of lncRNA biomarkers in distinguishing early-stage and advanced-stage patients, and the Chi-square test was used to test the significance of the association between tumor status and lncRNA biomarkers.

### Statistical analysis

To obtain optimal lncRNA biomarkers significantly associated with tumor progression, a random forest supervised classification algorithm was used to narrow down the number of lncRNAs by several iterative steps, in which one-third of the least important lncRNAs were discarded at each step according to their importance score using R packages “randomForest” as previously described [47]. Finally, optimal lncRNA biomarkers were integrated to construct a predictive risk classifier using support vector machine (SVM) with the sigmoid kernel. An unbiased performance estimate of SVM-based lncRNA risk classifier in distinguishing early-stage and advanced-stage patients was evaluated using 5-fold cross-validation strategy followed by establishing receiver operation characteristic (ROC) curve. The area under the receiver operating curve (AUC) was produced by plotting true positive rates (sensitivity) against false positive rates (1-specificity) to indicate the prediction performance. Kaplan-Meier survival curves and log-rank tests were used to assess the differences in survival time between the predicted early-stage-like group and advanced-stage-like patients using R packages “survival”.

### Functional enrichment analysis

Functional enrichment analysis of Kyoto encyclopedia of genes and genomes (KEGG) for mRNAs co-expressed with lncRNA biomarkers was performed to infer potential biological pathways of lncRNA biomarkers using DAVID Bioinformatics Tool (version 6.7) [48] limited to KEGG pathway categories. The biological pathways with a *p*-value of < 0.1 and an enrichment score of > 2.0 using the whole human genome as background were considered as a significantly enriched pathway.
